# Role of strategies used by young people for dealing with emotional distress: a qualitative study in deprived urban neighborhoods in Latin America

**DOI:** 10.1007/s44192-025-00143-3

**Published:** 2025-02-20

**Authors:** Isabela Osorio Jaramillo, Carlos Gomez-Restrepo, Luis Ignacio Brusco, Francisco Diez-Canseco, Catherine Fung, Karen Ariza-Salazar, Natividad Olivar, Mauricio Toyama, Diliniya Stanislaus Sureshkumar, José Miguel Uribe-Restrepo, Fernando Luis Carbonetti, Ana L. Vilela-Estrada, Stefan Priebe

**Affiliations:** 1 Department of Clinical Epidemiology and Biostatistics, Pontificia Universidad Javeriana, Bogotá, Colombia; 2 Department of Psychiatry and Mental Health, Pontificia Universidad Javeriana, Bogotá, Colombia; 3 Hospital Universitario San Ignacio, Bogotá, Colombia; 4 School of Medicine, Department of Psychiatry and Mental Health, University of Buenos Aires, Buenos Aires, Argentina; 5 Universidad Peruana Cayetano Heredia, CRONICAS Center of Excellence in Chronic Disease, Lima, Peru; 6 Unit of Social and Community Psychiatry, Queen Mary University of London, Wolfson Institute of Population Health, London, UK; 7 Centre for Psychosocial Medicine, University of Hamburg, Hamburg, Germany

**Keywords:** Young people, Coping, Social resources, Mental health, Emotional distress

## Abstract

**Background:**

Many young people achieve recovery from mental health problems by using strategies to manage emotional distress and enhance well-being. Given that little is known about the functions of these strategies, especially in Latin American countries, this study aims to describe the usefulness of the resources used by youth from deprived urban neighborhoods in Bogotá (Colombia), Buenos Aires (Argentina), and Lima (Perú) in managing emotional distress.

**Methods:**

112 in-depth interviews about strategies for dealing with emotional distress and their perceived function were conducted with young people from three Latin American cities. The sample included young adults and adolescents in a longitudinal cohort study. A thematic content analysis was carried out.

**Results:**

Participants identify different functions linked to their strategies to cope with emotional distress or increase their well-being. However, "coping with distress," "perceived support," and "distraction" are the three main functions of the strategies used by them in situations of emotional distress. Each of these functions comprises several aspects, some of which overlap between functions, such as motivation, emotional expression, companionship, and regulation.

**Conclusion:**

The variety of functions linked to the strategies used by young people to cope with emotional distress or enhance their well-being could guide the facilitation of a favorable social and interpersonal context through public policies and a community approach that promotes young people's access to strategies to cope with emotional distress.

**Supplementary Information:**

The online version contains supplementary material available at 10.1007/s44192-025-00143-3.

## Background

### Introduction

Mental health problems of young people and adolescents are a current global priority of the public health system [1, 2]. The presence of depressive symptoms or anxiety (emotional distress) is prevalent in this population; for 2019 in South Latin America, an age-standardized prevalence of 2777.3 cases for depressive disorders per 100,000 inhabitants was estimated (95%; 2492.5–3111.5) and 5125.8 cases for anxiety disorders per 100,000 inhabitants (95%; 4459.58–5885.1) [3] Sometimes, this emotional discomfort becomes chronic, while other young people and adolescents manage to overcome it, partly due to the support of the environment and the use of various strategies as sports, arts, educational or religious and spiritual activities or social support, understood as activities, attitudes or resources that allow them to face or avoid these situations [4].

To our knowledge, no studies have been developed in Latin America to explore the role of strategies used by young people to cope with emotional distress. It is essential to study young people's perceptions of why and what for they use these strategies, which help them recover or prevent states of emotional discomfort or maintain and improve a state of mental well-being. Identifying the underlying function or utility of these strategies allows for a greater understanding of them and eventually strengthens public mental health interventions and programs toward a more comprehensive approach to mental health in young people, which includes promotion, prevention, and recovery.

This study describes the usefulness function of the resources and strategies used by young people and adolescents from deprived urban neighborhoods in Bogotá, Buenos Aires, and Lima to manage emotional discomfort.

## Methods

### Study design

Between August 2022 and August 2024, A descriptive qualitative study was conducted in Bogotá (Colombia), Lima (Perú), and Buenos Aires (Argentina) using an exploratory approach. This approach enabled the identification and description of the unique experiences and perspectives of the youth participants [5]. This study was linked to the OLA Research Program (Building Resilience and Resources to reduce depression and Anxiety in Young People from Urban Neighborhoods in Latin America). The overall objective of the OLA Research Program is to identify characteristics, resources, and activities that help young people living in urban settings in Latin America to prevent or recover from depression or anxiety [6].

To achieve this objective, the OLA Research Program includes a cohort of adolescents aged 15–16 years and young adults aged 20 to 24 years (At the time of their enrollment in the research program), with symptoms of emotional distress (defined as those participants with scores > 9 on one or both scales for screening symptoms of depression and anxiety (Patient health questionnaire- PHQ-8 [7] and General Anxiety Disorder- GAD-7 [8] respectively). The participants of the OLA Research Program met additional inclusion criteria beyond the age groups previously mentioned, such as residing in the 50% most vulnerable areas of each city and the ability to provide informed consent. Young individuals with intellectual disabilities, a diagnosis of severe mental illness, and illiteracy were excluded. Sample details, recruitment strategies, and informed consent process, among others, are specified in previous publications [6].

### Participants

A sample of 112 participants from Bogotá, Buenos Aires, and Lima (40, 42, and 30, respectively) who completed the 12-month follow-up of the OLA Research Program and consented to participate in this activity at the time of registration, were selected by convenience. A balance of the sample was sought in terms of age group, gender, country of origin, and recovery status at the time of follow-up (those participants with scores < 10 on both scales for screening symptoms of depression and anxiety were considered recovered). As the 12-month follow-up was completed, a sufficient number of participants were invited to obtain the required sample in each country.

### Data collection

Two consecutive stages were developed: "Readiness" and "Development" for the data collection:

In Stage 1, "Readiness" researchers from each city created a guide of questions for the in-depth interviews, formulated as free conversations with determining elements [9]. This allowed the participants to offer information little by little about the strategies of young people in situations of emotional distress and explore their function.

This guide was evaluated in terms of understanding and relevance through a pilot interview exercise with seven young people aged between 15 and 24 years old (3 in Bogota, 2 in Lima, and 2 in Buenos Aires) from the Lived Experience Advisory Committee (LEAP) [6] and participants of the cohort of OLA Research Program or young people close to research assistants. The selected questions included the type of strategies or activities carried out in situations of emotional distress, the why and frequency of use, and details regarding their perceived function and situations of their use.

A list of resources was used to facilitate the conversation, composed of specific strategies or activities (for example, talking with friends, talking with family, doing sports or physical activity, doing artistic or creative activities, surfing the Internet, among others). This allowed exploring a wide range of strategies commonly used by young people, according to the findings of the first phase of the study [10].

Stage 2."Development": Between August 2022 and January 2024, a team of eight trained professionals (1 sociologist in Colombia, 2 psychologists and 2 psychiatrists in Argentina, and 3 psychologists in Peru) developed in-depth interviews remotely, using platforms for virtual meetings such as Microsoft Teams, Zoom, or Google Meet. All interviews were conducted individually and in Spanish (the native language of the researchers and participants) following a topic guide. Each interview had an average duration of 38 min, with a range between 13 and 140 min.

All interviews were audio recorded and transcribed verbatim, ensuring data anonymization. For this purpose, a combination of transcription mechanisms was used: in Colombia and Argentina, initial transcription supported by artificial intelligence was used, and a manual review was carried out for error debugging. In Peru, manual transcription was carried out.

### Data analysis

For this study, a thematic content analysis based on inductive coding [11] was carried out. Then, the codes were tentatively deduced step by step, with continuous "feedback" until the main categories were obtained [11]. Three stages were developed: "Inductive coding", "Coding and feedback of the codebook", and "Data analysis".

In Stage 1, "Inductive Coding," a group of seven researchers selected six interviews for convenience (2 from each country considered by each team with sufficient data richness) to familiarize themselves with the information and free-code the emerging themes based on the strategies used by young people against emotional distress using the NVivo version 14 software [12].

Subsequently, in August 2023, in a face-to-face meeting, the research team organized and categorized the identified information and generated a codebook with seventeen functions linked to the 31 strategies used by young people (identified and grouped into nine groups of strategies during the same process of creating the codebook) for a total of 510 combinations or codes on the function of the strategies, to guide the coding of the entire of the interviews.

Stage 2, "Data analysis: coding and codebook feedback”: between August 2023 and January 2024, eleven members of the research team (4 in Argentina, 3 in Colombia, and 4 in Peru) participated in coding all of the study interviews using the established codebook and periodically discussing new emerging codes and other adjustments to the codebook. Coding was performed using Nvivo 14 software [12].

Stage 3. "Thematic content analysis": between January 2024 and August 2024 the final stage of the analysis process was conducted. Initially, the qualitative database of each country hosted in the Nvivo 14 software was reviewed and cleaned. Two members of the Colombian research team grouped and downloaded the study data into Microsoft Excel and prepared frequency tables with the number of mentions for each identified function and the number of participants who mentioned each function to facilitate the identification of patterns in the data and guide the thematic content analysis.

Finally, at the second level of the thematic content analysis, three of the total number of functions were selected according to the highest number of mentions among the study participants (those with several mentions greater than 75 were taken) and were subsequently identified, regrouped and analyzed by themes [13]. This classification allowed, on the one hand, to explore in detail the content of each of the three most mentioned functions and, on the other hand, to identify a series of common elements in the functions of the strategies [14].

## Results

### Characteristics of the participants

A total of one hundred and twelve interviews were conducted, forty-eight with adolescents and sixty-four with young adults from Bogotá, Buenos Aires, and Lima (40, 42, and 30, respectively). 61 female, 50 male, and one non-binary gender (Table 1).

**Table 1:** Sociodemographic characteristics of the participants

Recovery group*		Recovered			Not recovered		
Age group	Gender	Argentina	Colombia	Peru	Argentina	Colombia	Peru
Adolescents	Female	4	5	4	6	6	3
	Male	2	5	3	0	5	4
	Non-binary	0	0	0	0	0	1
Young adults	Female	9	5	3	7	4	5
	Male	9	4	4	5	6	3
	Non-binary	0	0	0	0	0	0
Total by country		24	19	14	18	21	16
Total according to recovery group		57			55		
Total interviews		112					

*Recovery group: indicates recovery status at 12-month follow-up. (those participants with scores < 10 on both scales for screening symptoms of depression and anxiety (GAD-7 and PHQ-8, respectively) were considered recovered)

Only 111 interviews were included in the analysis (In the interview of a recovered young adult man from Argentina, questions about the functions of the reported strategies were not addressed).

### Strategy functions

We grouped the functions of the strategies used by young people in situations of emotional distress into sixteen functions identified during the data analysis (Table 2).

**Table 2:** Description of functions of the strategies used to overcome emotional distress

Function	Description
Coping with problems	It encourages problem-solving or the search and reflection on alternative solutions
Coping with discomfort or the emotional impact of discomfort	It helps reduce, suspend, or eliminate the emotional impact of discomfort, even temporarily, including self-regulation and relaxation
Search or practice of healthy habits	Helps improve quality of life through sustained use over time
Connection with the present moment	Helps connect with the present moment or mindfulness (according to the explicit information provided by the young people)
Develop social skills	It promotes social interaction or increases the perception of one's ability to socialize
Enjoyment	It enables the experience of feeling pleasure, joy, or having a pleasant moment
Enjoy with others	Allows the experience of well-being due to sharing with others
Distraction	It allows you to mentally distance yourself from problems, worry or emotional discomfort
Emotional expression	Allows emotional expression and relief
Introspection	It allows the observation of one's internal world and its context, to understand one's reality, the actions taken or the feelings
Productive occupation of time	It allows you to perceive the use of time
Have another perspective	It helps to redefine one's own experience from seeing, listening to, or receiving testimonies, recommendations, and/or experiences from third parties
Perception of support	Provides a feeling of support, accompaniment, identification, or understanding from peer groups, family members, pets, etc
Personal fulfillment	Supports the fulfillment of goals, life projects, or personal development
Rest	Helps the participant take a break from discomfort
Optimistic vision	It allows you to have a positive vision or perspective of your emotional state, the future, or different situations and/or experiences

In general, the data disclosed a wide range of functions of the different strategies used by young people in emotional distress (the strategies will be described in detail in another publication). The study participants attributed between 9 and 16 functions to the different groups of strategies explored (Physical activity and sport: 15 functions; Artistic activities: 14; relaxation, self-regulation, and rest activities: 13; Educational activities: 9; spiritual and religious activities: 11; recreational and leisure: 16; personal resources: 15; social resources: 10; *for more information see supplementary material*).

### Description of main functions

The data present a high heterogeneity in the function of the strategies explored, covering between 20 and 497 mentions for each function. However, it is evident that coping with discomfort, distraction, and the perception of support are the functions with the most significant number of mentions and were mentioned by the greatest number of participants (Table 3), suggesting a higher level of importance over the other functions.

**Table 3:** Frequency of function responses by number of participants

Recovery group*	Non-recovered		Recovered		Total	
Functions	# Mentions	n	# Mentions	n	# Mentions	n
Coping with problems	54	22	61	24	115	46
*Coping with discomfort or the emotional impact of discomfort*	250	44	246	43	496	87
Search or practice of healthy habits	4	4	16	11	20	15
Connection with the present moment	14	7	10	6	24	13
Develop social skills	3	3	18	10	21	13
Rest	15	6	23	12	38	18
Enjoyment	57	27	68	23	125	50
Enjoy with others	26	14	38	22	64	36
*Distraction*	233	48	264	51	497	99
Emotional Expression	73	31	92	36	165	67
Introspection	35	19	50	20	85	39
Productive occupation of time	5	4	25	17	30	21
*Perception of support*	111	37	174	42	285	79
Personal Fulfillment	48	24	68	28	116	52
Have another perspective	66	30	68	29	134	59
Optimistic vision	65	28	55	28	120	56
**Total**	1059	54	1276	57	2335	111

*Recovery group: indicates recovery status at 12-month follow-up. (those participants with scores < 10 on both scales for screening symptoms of depression and anxiety (GAD-7 and PHQ-8, respectively) were considered recovered)

Through an approach to the thematic content of "coping with discomfort or emotional impact of discomfort", "distraction", "and perception of support", we identified recurring themes that described each function in detail and showed that some of these aspects are common among the functions. (As is illustrated in standard colors in Table 4).

**Table 4:**
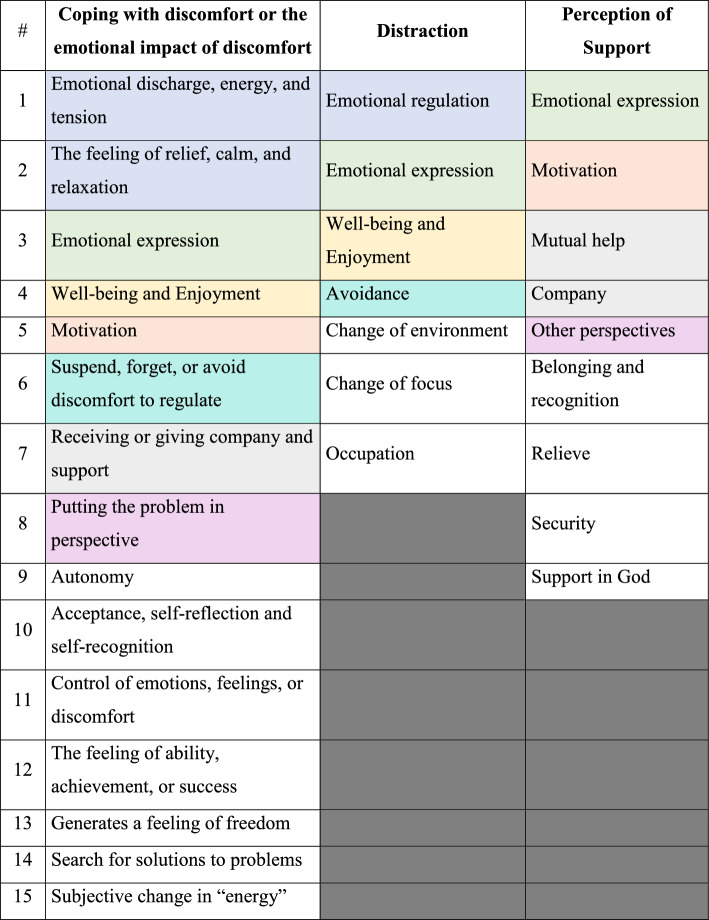
Themes for a description of the main functions

#### Coping with discomfort

Regarding the function of the strategies related to coping with discomfort, we found 15 recurring themes, which are linked to all the groups of strategies explored, but to a greater extent to Relaxation, self-regulation and rest activities, Recreational and leisure activities, and social resources:

Acceptance, self-reflection, and self-recognition: These frame young people's perceptions related to the possibility of recognition and acceptance of emotions, situations, or problems, in addition to reflection for greater mental clarity and thus reduce, suspend, or eliminate the impact of emotional discomfort.

*I think everything is like a process of acceptance, yes, because there is a part in which they talk a lot about the topic of avoiding. One thing that I did a lot before was how to avoid my emotions a lot (…) rather instead of doing what I normally used to do in those moments, which was like extending the situation and making it worse, therefore I used the tools that they taught me to simply diminish what I am feeling… (Female, adolescent, not recovered, Colombia).*

Autonomy: Reflects the usefulness mentioned by young people about some strategies, which allow them to learn to value spaces alone to face emotional discomfort, in addition to having clear priorities and routines and acting on them.

Search for solutions to problems: Young people relate this topic to managing situations or conflicts, allowing them to search for solutions, deal more appropriately, or finally disintegrate the problems.

Subjective change in "energy": Refers to experiences of activation or change in mood or environment, which are usually accompanied by a feeling of greater energy and connection with one's environment.

*I don't know if it's particularly severe anxiety, but I like to get up when I need to change my vibe and my energy because it's like a moment of relaxation. (Female, young adult, not recovered, Argentina).*

Control of emotions, feelings, or discomfort: This topic demonstrates that with specific strategies, young people perceive that they can manage emotions and impulses to avoid overwhelming feelings and reduce the intensity of feelings or emotional discomfort.

*When I feel anxious, meditation helps me focus on my breathing and disconnect from my worries. I can calm my mind and reduce the intensity of my emotions. It helps me see the situation differently and try to see how I solve it. (Male, young adult, not recovered, Argentina).*

Emotional discharge, energy, and tension: This category reflects young people's perceptions regarding the possibility of downloading negative energy, venting, clearing the mind, relaxing, and releasing burdens with some strategies. In everyday language, it overlaps with the phrase "it is cathartic or liberating."

*It helps me release my emotions. And sometimes when you have a specific emotion and it's kind of deep inside you, it's like you only focus on that. You don't let other things, no matter how good they are, cover that emotion that happens to you. And sometimes tell someone else or hear that someone else is going through the same thing. That, as I told you, I am not alone on this path and it comes to releasing the emotion or the tension of the emotion. (Female, young adult, not recovered, Argentina).*

Well-being and Enjoyment: According to young people's perceptions, some strategies promote feelings of fun, joy, and comfort in a hedonic dimension, aligned with coping with discomfort or seeking emotional well-being.

Emotional expression: Young people's perceptions frame aspects such as being able to express themselves freely about emotions and situations or speak with more confidence regarding emotional discomfort.

*Well… I feel like I can tell him everything I'm feeling and he gives me that confidence to get everything out there, so I tell him what I think, with the situations I'm going through and now eh… I stopped feeling suffocated by those feelings and everything I was saving… (Female, adolescent, recovered, Peru).*

Generates a feeling of freedom: It frames what young people expressed regarding the fact that some activities make them feel free about other people, situations, spaces, or uncomfortable emotions.

Motivation: It refers to the possibility that young people find to develop or increase personal interests or purposes when carrying out some activities. It is closely related to things that raise their spirits, increase well-being, or suspend emotional discomfort.

*Talking to my mother, I think that listening to my mother gives me the strength to continue and to set goals for tomorrow despite how badly one is doing or what is missing. (Male, young adult, recovered, Argentina).*

Putting the problem in perspective: Refers to using some strategies to transform negative judgments about difficult experiences and see issues from other points of view.

Receiving or giving company and support: Young people frame this topic in mutual help, giving or receiving advice and motivation in the face of discomfort, feeling supported and accompanied, or feeling that someone can listen to them about their emotions, situations, or problems, as an opportunity to cope with discomfort.

*Feeling that at some point they can understand me or something like that and understand what is happening to me. Maybe between my brothers and my mother, it helps them know how I am. And that I understand in some way how they can help me. (Female, adolescent, not recovered, Argentina).*

The feeling of relief, calm, and relaxation: One of the most significant themes in the function of strategies linked to coping, it frames aspects such as calming down, soothing, or being relieved, in addition to feeling peace, well-being, de-stressing, and relaxation in situations of discomfort.

*No, I think it is something that I like, and I feel that it is like a part that relaxes my body and my mind because it is like being in another place that makes you feel happy… (Female, adolescent, not recovered, Colombia).*

The feeling of ability, achievement, or success frames the perception of dealing with bad situations or negative emotions and having the feeling of achieving or achieving something successfully, strengthening self-efficacy through some strategies.

Suspend, forget, or avoid discomfort to regulate: Characterizes aspects such as avoidance, disconnection from reality, forgetting everything, distracting oneself, pausing or suspending discomfort, or finally slowing down moments of emotional discomfort to balance emotions.

*…However, during the game, it was like… eh… I relaxed, I relaxed, I was like that… it's like… not thinking about anything, practically being in another world… (Male, adolescent, recovered, Peru).*

#### Distraction

The content of the information reported by young people regarding the function of strategies associated with distraction in situations of emotional distress is grouped into seven recurring themes, which are mainly linked to recreational and leisure activities and physical and sporting activities, although this function is mentioned in almost all of the strategies, except spiritual and religious activities:

Well-being and Enjoyment: This refers to finding spaces of enjoyment, fun, or opportunity in distracting activities. It is the opportunity to take refuge or do the activities preferred by young people to feel better and raise their spirits instead of facing or thinking about the situation, which generates discomfort.

*Why do you think talking with friends helps you feel better? _Interviewee: Because they always find a way to make me laugh and forget, even at that moment, the problem I have. That helps me a lot, too. (Female, adolescent, not recovered, Argentina).*

Change of environment: Young people report that some strategies allow them to change their environment or energy, in addition to the sensation of entering realities other than the situation or emotional discomfort.

*What most people say, it kind of… it transports you to another world, so to speak, so I immerse myself in that world and listen to music, making me feel better. (Male, adolescent, not recovered, Colombia).*

Change of focus: It is related to the possibility of concentrating or focusing all their attention on activities that they like or that allow them to experience more mental clarity and learn something new, to temporarily deviate from problems or discomfort, and in this way, to downplay the importance of themselves.

*(…) When I was doing my exercises, running, jogging, I don't know, I wasn't thinking about anything else, but I just felt like maybe a physical pain. So that pain and tiredness helped me clear my head, you know? Like thinking about other things and not overthinking the things that I was already in, well… starting to go down, so to speak. (male, adult, not recovered, Colombia).*

Avoidance encompasses the opportunity to get rid of problematic situations or emotional discomfort through the use of some strategies. It is described as disconnecting from reality, moving away from or suspending negative problems, emotions, or feelings, distracting the mind, escaping, forgetting, or evading the problem, worrying about the future, and overthinking situations.

*Yes, there are other things I sometimes do when I feel anxious, but I started now. A while ago, I started going out alone for a run. I had never done it before, and I realized that it is good because it helps me not to think, to clear my mind of worries, to focus on the path, it is almost like a walk. And I realized that in addition to maintaining myself physically, it changes my mood. (male, adult, not recovered, Argentina).*

Emotional expression: Refers to strategies used to distract themselves and allow young people to express their problems.

Emotional regulation: Refers to distraction as an opportunity to clear the mind, discharge energy, release emotions, relax, or relieve emotional discomfort by carrying out activities that distract from problems.

*Well no, he doesn't think, I think you clear your mind, right? I feel that this is clearing your mind too much, when you need to do physical activity, exercise, to feel healthy because it is like clearing your mind. You are like taking away, let's say, you are at that moment, you are thinking about exercising, about even feeling tired; to say, well, I'm going to ask so many questions, but that helps you clear your mind a little from the other things that you have around you. (female, adult, recovered, Colombia).*

Occupation: Refers to the opportunity to occupy one's time or mind, forget about problems, and entertain oneself in moments of emotional discomfort. Likewise, young people reported that some strategies allow them to reduce the feeling of loneliness or help time pass faster.

*In some ways, I like languages, so when I have felt bad because of a problem, whether academic or sentimental, getting into studying languages has helped me a lot. Let's say you keep your mind busy with something you really like. (Female, adult, not recovered, Peru).*

#### Perception of support

The function of strategies related to the perception of support in situations of emotional distress is summarized in 10 recurring themes, linked mainly to two groups of strategies of the nine explored: Spiritual and religious activities and Social resources:

Relief: Frames the possibility offered by some strategies to reduce pressure and experience happiness, peace, or the subjective experience of feeling good when perceiving yourself accompanied or supported in situations of emotional discomfort.

Support in God: Young people identify that activities related to prayer and connection with God allow them to have a subjective experience of support, confidence, and relief in the face of problems or emotional discomfort.

Mutual help: Some strategies allow young people to receive advice, guidance, or words of encouragement or experience the feeling of being helped, supported, or supported emotionally, which increases their well-being and makes it possible for them to face emotional distress.

*Talk to my friends, because they know me, they know what I am, sometimes the same things happen to us, so we all help each other. (Female, adolescent, recovered, Argentina).*

Company: Indicates that some strategies linked to the perception of support generate the certainty of being able to tell and talk with others about situations of emotional discomfort and other experiences. It also includes the opportunity to feel accompanied and listened to empathetically.

Emotional expression: The perception of support is linked to strategies for being heard and freely expressing and sharing emotions, situations, and experiences.

*I feel like I can tell him everything I'm feeling and he gives me that confidence to get everything out, so I tell him what I think, about the situations I'm going through and I stop feeling suffocated by those feelings and everything I was keeping in, and… Feeling listened to has helped me, I feel supported, so to speak, and now… I don't feel ignored (Female, Adolescent, Recovered, Peru).*

Motivation: Some strategies allow young people to feel motivated to move forward, lift their spirits, or overcome emotional discomfort from the trust (strength) that others have in them.

Other perspectives: Frames what young people say about seeing situations differently by receiving or finding other versions or opinions about the experiences.

Belonging and recognition: Linked to the opportunity that young people have to feel understood, identified with others or part of something, recognized, validated, and loved by using some strategies.

*I think that when I feel more loved. I feel that the support and, more than anything, quality time with people, helps me a lot because I am afraid of feeling alone and being alone in general. (Female, Adolescent, Not recovered, Colombia).*

Security: refers to the perception of support as an opportunity for young people to feel sheltered or protected in situations of emotional discomfort, and to be safe, comfortable, or confident in the open expression of their discomfort or problems.

*I can tell him what's happening to me, I can be completely honest, I know he will understand me and not criticize me. Or, if you don't like something, you'll tell me. (Male, adolescent, recovered, Argentina).*

## Discussion

As a result of this study, sixteen functions were identified for the nine groups of strategies used by young people in situations of emotional distress: "Coping with problems", "Coping with Discomfort or the emotional impact of discomfort", "Search or Practice of healthy habits", "Connection with the present moment", "Develop social skills", "Enjoyment", "Enjoy with others", Distraction", "Emotional expression", "Introspection", "Productive occupation of time", "Perception of support", "Personal fulfillment", "Rest", "Have another perspective" and "Optimistic vision".

This diversity of functions reveals: a. the diversity of options that young people find to cope with situations of emotional distress; b. the variety of functions associated with each strategy; and c. the overlap in the functions that some strategies share between them.

The findings of this study also point to the presence of common elements or themes in which the aspects that the young people used to describe each function were grouped, such as motivation, emotional expression, companionship, and regulation. This suggests that young people recognize a diverse range of opportunities rather than limited ways to address mental health. In turn, it indicates no specific relationship between a strategy and its function.

Coping with discomfort, distraction, and the perception of support were the functions most frequently reported by the young people when referring to the role of different coping strategies used in situations of emotional discomfort, which is supported by evidence on the various styles focused on the problem or on emotion, which involve aspects such as seeking social support, moderating stressful situations or planning, through actions and thoughts aimed at problem-solving or emotional regulation, such as distancing, seeking social support, and confrontation, among others [15–17].

Functions such as coping with discomfort, emotional expression, and introspection align with elements of emotion-focused coping presented in other studies [15], or represent elements similar to those promoted in therapeutic interventions, and this reiterates how non-clinical strategies, but called "therapeutic" by young people, strengthen and develop people's capacities and abilities in dealing with emotional discomfort. For example, studies have shown that some artistic interventions in young people are therapeutic in that they are a "liberating means of expression [18]; they encourage the creation of socialization networks and with them positive responses from the environment, social contact, support networks, participation, and various motivations towards positive aspects [19–21].

Other functions, identified in our study as the search or practice of healthy habits, developing social skills, enjoyment and enjoyment with others, productive occupation of time, and the perception of support, highlight the role that a favorable environment plays in the access to strategies which promote the perception of benefits or usefulness of the strategies. Some studies have shown that young people believe that a mental health-friendly city should provide them with the skills, opportunities, and places necessary to build and maintain healthy social relationships with their peers, between generations, and as members of a community [10, 22, 23].

Conversely, some studies revealed that the built environment is seen as a potential resource, so differential access to these resources can exacerbate mental health inequalities. Since the perception of the neighborhood as dangerous in turn influences adolescents' mental health: the more threatening the neighborhood, the more frequent are symptoms of depression, anxiety, oppositional defiant disorder, and conduct disorder [24, 25].

In this sense, a community approach to health that promotes enriched environments with quality infrastructure and services available for access to sport, culture, education, and entertainment could facilitate young people's access to activities and resources for leisure and use of free time, as promoted by national and international policies around mental [2], taking advantage of the potential function of these strategies for care and recovery of well-being.

## Limitations

This study contributes to the exploration of young people's resources to overcome mental distress in large urban settings in three Latin American countries and provides an understanding of young people's perceptions of the benefits or functions of the different strategies they use in situations of emotional distress. However, because it is not a representative sample, it is not possible to extrapolate the results. To young Latin Americans. Socio-environmental, cultural, and personal conditions are determinants of young people's mental health, access, recognition, and use of different strategies.

Secondly, given that the semi-structured interviews were carried out at a stage after the COVID-19 pandemic, it cannot be ruled out that the residual social and emotional impact has modified the access, preferences, and perceptions of young people regarding the strategies and their function to help them face situations of emotional distress.

Finally, exploring behaviors and opinions about the function of strategies only explains the mechanisms perceived by young people subjectively. More extensive research is required to study the associations and effects of different strategies or resources on emotional distress.

## Conclusions

This research shows that the young people in the study attribute a wide range of functions linked to the various strategies used in the face of emotional distress and well-being situations. Common themes were identified within the most representative functions (coping with discomfort, distraction, and perception of support) that suggest no intrinsic relationship between the strategies and their functions. On the contrary, the function is directly related to the subjectivity of the young person who recognizes and activates various strategies to recover or maintain and improve mental well-being.

Recognition of these functions could guide the facilitation of a favorable social and interpersonal context through public policies and a community approach that promotes young people's access to strategies to cope with emotional distress.

## Supplementary Information

Below is the link to the electronic supplementary material.

Supplementary material 1.

## Data Availability

The consent forms did not specify that the data would be deposited in a public repository, so we cannot deposit the data. The deidentified participant dataset will be made available from the corresponding author () upon reasonable request and subject to a data-sharing agreement.
