# Supplementary material for: Role of strategies used by young people for dealing with emotional distress: a qualitative study in deprived urban neighborhoods in Latin America
Source: Discov Ment Health. 2025 Feb 20;5(1):14. doi: 10.1007/s44192-025-00143-3 (PMC11842665; doi:10.1007/s44192-025-00143-3)
Supplement: Supplementary file 1 — Supplementary material 1. [file 44192_2025_143_MOESM1_ESM.docx]

Role of strategies used by young people for dealing with emotional distress: A qualitative study in deprived urban neighborhoods in Latin America.

**Supplementary material**

**Table: Functions according to strategies**

| **Functions of the strategies according to the number of subjects who mentioned each function (n=111)** | **Physical activity and sports** | | **Artistic activities** | | **Relaxation, self-regulation, and rest activities** | | **Educational activities** | | **Spiritual and religious activities** | | **Recreational and leisure activities** | | **Personal resources** | | **Social resources** | | **Mental health services** | |
| --- | --- | --- | --- | --- | --- | --- | --- | --- | --- | --- | --- | --- | --- | --- | --- | --- | --- | --- |
|  | **n** | **%** | **n** | **%** | **n** | **%** | **n** | **%** | **n** | **%** | **n** | **%** | **n** | **%** | **n** | **%** | **n** | **%** |
| Coping with problems | 2 | 2% | 1 | 1% | 10 | 9% | 0 | 0% | 2 | 2% | 6 | 5% | 19 | 17% | 16 | 14% | 7 | 6% |
| Coping with discomfort or the emotional impact of discomfort | 26 | 23% | 22 | 20% | 35 | 31% | 2 | 2% | 12 | 11% | 42 | 38% | 33 | 29% | 26 | 23% | 16 | 14% |
| Search or practice of healthy habits | 12 | 11% | 0 | 0% | 1 | 1% | 0 | 0% | 0 | 0% | 1 | 1% | 1 | 1% | 0 | 0% | 1 | 1% |
| Connection with the present moment | 4 | 4% | 2 | 2% | 5 | 4% | 0 | 0% | 1 | 1% | 2 | 2% | 3 | 3% | 1 | 1% | 0 | 0% |
| Develop social skills | 2 | 2% | 1 | 1% | 0 | 0% | 0 | 0% | 1 | 1% | 5 | 4% | 2 | 2% | 5 | 4% | 1 | 1% |
| Rest | 1 | 1% | 0 | 0% | 11 | 10% | 0 | 0% | 0 | 0% | 7 | 6% | 0 | 0% | 1 | 1% | 0 | 0% |
| Enjoyment | 12 | 11% | 10 | 9% | 4 | 4% | 4 | 4% | 2 | 2% | 33 | 29% | 2 | 2% | 9 | 8% | 0 | 0% |
| Enjoy with others | 8 | 7% | 1 | 1% | 0 | 0% | 0 | 0% | 0 | 0% | 16 | 14% | 1 | 1% | 20 | 18% | 0 | 0% |
| Distraction | 36 | 32% | 23 | 21% | 11 | 10% | 11 | 10% | 0 | 0% | 79 | 71% | 17 | 15% | 30 | 27% | 2 | 2% |
| Emotional expression | 3 | 3% | 14 | 13% | 4 | 4% | 2 | 2% | 9 | 8% | 9 | 8% | 3 | 3% | 42 | 38% | 7 | 6% |
| Introspection | 1 | 1% | 4 | 4% | 7 | 6% | 0 | 0% | 6 | 5% | 10 | 9% | 21 | 19% | 8 | 7% | 9 | 8% |
| Productive occupation of time | 9 | 8% | 2 | 2% | 0 | 0% | 7 | 6% | 0 | 0% | 1 | 1% | 4 | 4% | 1 | 1% | 0 | 0% |
| Perception of support | 0 | 0% | 2 | 2% | 1 | 1% | 1 | 1% | 17 | 15% | 7 | 6% | 5 | 4% | 72 | 64% | 8 | 7% |
| Personal fulfillment | 13 | 12% | 6 | 5% | 1 | 1% | 16 | 14% | 5 | 4% | 13 | 12% | 17 | 15% | 8 | 7% | 3 | 3% |
| Have another perspective | 1 | 1% | 1 | 1% | 4 | 4% | 1 | 1% | 1 | 1% | 8 | 7% | 11 | 10% | 43 | 38% | 13 | 12% |
| Optimistic vision | 2 | 2% | 2 | 2% | 9 | 8% | 1 | 1% | 6 | 5% | 11 | 10% | 39 | 35% | 16 | 14% | 0 | 0% |
| **Total number of functions (N=16)** | 15 | | 14 | | 13 | | 9 | | 11 | | 16 | | 15 | | 15 | | 10 | |
